# Hydrophobic Porous Liquids with Controlled Cavity Size and Physico‐Chemical Properties

**DOI:** 10.1002/advs.202305906

**Published:** 2023-11-30

**Authors:** Lorianne Ginot, Amal El Bakkouche, Fabrice Giusti, Sandrine Dourdain, Stéphane Pellet‐Rostaing

**Affiliations:** ^1^ ICSM, CEA, CNRS, ENSCM Univ Montpellier Marcoule 30207 France

**Keywords:** hydrophobicity, nanoscale ionic materials, porous liquids, hollow silica spheres, solvent extraction

## Abstract

Developing greener hydrometallurgical processes implies offering alternatives to conventional solvents used for liquid‐liquid extraction (LLE) of metals. In this context, it is proposed to substitute the organic phase by a hydrophobic silica‐based porous liquid (PL). Two different sulfonated hollow silica particles (HSPs) are modified with various polyethoxylated fatty amines (EthAs) forming a canopy that provides both the targeted hydrophobicity and liquefying properties. This study shows that these properties can be tuned by varying the number of ethylene oxide units in the EthA: middle‐range molecular weight EthAs allow obtaining a liquid at room temperature, while too short or too long EthA leads to solid particles. Viscosity is also impacted by the density and size of the silica spheres: less viscous PLs are obtained with small low‐density spheres, while for larger spheres (c.a. 200 nm) the density has a less significant impact on viscosity. According to this approach, hydrophobic PLs are successfully synthesized. When contacted with an aqueous phase, the most hydrophobic PLs obtained allow a subsequent phase separation. Preliminary extraction tests on three rare earth elements have further shown that functionalization of the PL is necessary to observe metal extraction.

## Graphical abstract

Porous fluids are promising candidates for applications because they combine high porosity and the advantages of liquid manipulations. To be used in metal extraction, they need to be hydrophobic and contain large pores. A series of silica‐based porous liquids with adjustable porosity and hydrophobicity is proposed together with a proof of concept of liquid‐liquid extraction of metals.

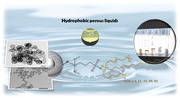

## 1 Introduction

Liquid‐liquid extraction (LLE) is a widely used separation technique for many applications, ranging from analytical methods to water treatment or industrial hydrometallurgical processes.^[^ ^1^ ^]^ In this latter case, LLE is the most common technique applied since it can handle large amounts of metals, by generally employing low‐cost compounds. For instance, it is largely applied in the recycling chemistry of critical metals such as rare earth elements from permanent magnets.^[^ ^2^ ^]^ Besides, liquids are far simpler to manipulate and store than gas or solids. LLE unfortunately implies the use of huge amounts of solvents and diluents, thus having a high environmental cost,^[^ ^1^, ^2^ ^]^ and it also faces the well‐known drawback of the formation of a third phase.^[^ ^3^ ^]^ Therefore, alternatives are required, and a number of studies are focusing on new eco‐friendly formulations^[^ ^1^, ^4^ ^]^ or non‐volatile organic phases like ionic liquids or deep‐eutectic solvents.^[^ ^5^, ^6^, ^7^ ^]^ Flotation is also considered to develop new routes of separation.^[^ ^8^ ^]^ Solid‐liquid extraction is usually proposed as an alternative to replace the solvent phase by a porous solid capable of extracting the elements of interest generally with excellent capacity and selectivity.^[^ ^3^, ^9^, ^10^, ^11^ ^]^ Despite these good performances, the use of solids requires to change infrastructure with respect to already existing ones. Given that they combine the properties of porous solids with the easiness of manipulating liquids, porous liquids might be good candidates as an alternative to the solvent phase in LLE.

Porous iquids (PLs) have been a fast‐growing field of research since their concept was first introduced by O'Reilly et al.^[^ ^12^ ^]^ in 2007. They describe liquid materials at room temperature or below 100 °C that have an intrinsic porosity, due to the presence of host cage molecules, particles, or networks, either dispersed in a hindered solvent, chemically modified, or physically treated to ensure a liquid behavior through weak interactions. This intrinsic porosity is sustainable and significant enough to consider these materials as carriers for many species. Despite their high potential, the application of PLs is consistently restricted to gas sorption so far.^[^ ^13^, ^14^, ^15^, ^16^ ^]^ In fact, among the different kinds of PLs that exist, most of them contain cavities less than 1 or 2 nanometers large, implying that the pores would be too small to welcome larger species like metal ions or metalorganic particles. In contrast, silica‐based PLs are promising materials for metal extraction or catalysis because of their low cost, tunable and relatively easy synthesis, and the nanometric size of their cavities.^[^ ^14^, ^17^ ^]^ Hence, Hemming et al.^[^ ^18^ ^]^ recently demonstrated the possibility of encapsulating nanoparticles of gold, platinum, and palladium inside the silica hollow cores during their synthesis, without significantly affecting the grafting steps forming the PL afterward. Furthermore, Yang et al. successfully separated heavy metals like lead and copper from a water phase by using a magnetic silica‐based PL to make the collection possible.^[^ ^19^ ^]^ In addition, at variance with LLE requiring the use of high amount of toxic volatile solvents, solvent‐free silica‐based PLs account for a process with low impacts on the environment.

Silica‐based PLs were synthesized for the first time by Zhang et al.^[^ ^14^ ^]^ in 2015. This synthesis consists in grafting a cationic organosilicon to the surface of hollow Silica Particles (HSPs), then adding a second layer of a sulfonated polyethylene glycol (PEG) derivative bound by ionic interactions. This last step is responsible for the liquid behavior of the porous material, along with its hydrophilicity, and no additional solvent is required. Many of the latest studies reported in the literature are restricted to systems based on Zhang's method,^[^ ^15^, ^20^, ^21^, ^22^, ^23^ ^]^ and only few knowledge has been gathered about their synthesis and their tunable properties up to now. Very recently, Ben Ghozi Bouvrande et al.^[^ ^23^ ^]^ assessed various experimental conditions for the synthesis of silica‐based PLs and highlighted the impact of different parameters (such as temperature and emulsion formulation) on PLs final properties. Meanwhile, Lai et al.^[^ ^24^ ^]^ investigated the impact of the grafting density and the molecular weight of the tethered groups on the permeability and the viscosity of the PL.

Considering the numerous applications involving HSPs described in the literature, it would be worthy to consider their extension to silica‐based PLs. Hence, the fabrication of stable antibacterial coatings,^[^ ^25^ ^]^ functional superhydrophobic surfaces,^[^ ^26^, ^27^, ^28^ ^]^ smart inks and paints,^[^ ^29^, ^30^ ^]^ and functional glasses, as well as catalysis in hydrophilic or hydrophobic media,^[^ ^18^, ^31^ ^]^ cosmetics,^[^ ^32^ ^]^ and LLE are all applications that could fit to these innovative liquid materials, along with gas sorption and separation. To broaden the applications of PLs, it is necessary to adapt the synthesis to tune their structural and physicochemical properties. For instance, LLE requires a hydrophobic PL, as well as adjustable and functionalizable silica cores. In particular, the cavities of the HSPs used by Zhang et al. are small (14 nm of internal diameter) whereas syntheses of bigger HSPs with larger pores inside the shell are largely described in literature (with internal diameters over 100 nm).^[^ ^27^, ^33^, ^34^, ^35^ ^]^ Forming a porous liquid with such big cavities could lead to an even broader range of applications. In particular for LLE, a bigger cavity could mean more room for a possible metallic nucleation, while larger pores could facilitate the diffusion of metallic ions inside the shell.

**Figure 1:**
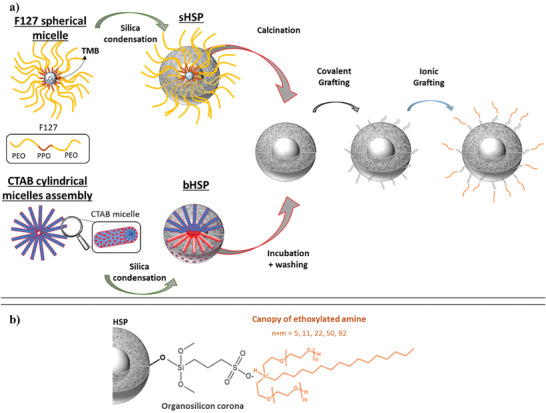
Synthesis of the porous liquids. a) The main four steps of the synthesis: HSP formation, calcination, covalent grafting of the sulfonated organosilicon, and ionic binding with the ethoxylated fatty amine. b) Scheme of the final grafting at the HSP surface. For the sake of simplification, a generic structure of commercially available modified tallow amines was given as polyethoxylated derivatives of hexadecylamine. The overall number of ethylene oxide units (n+m) was varied from 5 to 92.

This study proposes a new route to synthesize hydrophobic PLs with adjustable size of cavities. As illustrated in **Figure** **1**a, PLs with various particles size, density, and porosity were prepared: small non‐porous silica nanoparticles (SiNPs) and porous HSPs with two different sizes (“small” HSPs, which are few tens of nanometers large, referred to as sHSPs, and “big” HSPs, which are few hundreds of nanometers large, referred to as bHSPs) were compared. Inspired from the work of Rodriguez et al.,^[^ ^36^ ^]^ the particles were then covalently grafted by a sulfonated organosilicon. These modified silica spheres were further liquefied by the neutralization of the sulfonated surfaces with commercially available *N,N*‐polyethoxylated fatty amines (EthAs) forming the organic canopy (Figure 1b). The length of the hydrocarbon tail of these amphiphilic amines derivatives was considered as sufficiently hydrophobic and was fixed (mixture of C_16_ to C_18_ alkyl chains) whereas the length of the two PEG substituents was varying with a total number of ethylene oxide units (n+m) ranging from 5 to 92, allowing to tune the hydrophobicity of the final material.

Before achieving the synthesis of the corresponding PLs, the silica spheres were synthesized and characterized in terms of shape, size, density, and porosity. The physico‐chemical properties of PLs were also determined (physical state, melting temperature, viscosity, and molecular structure). Given that hydrophobicity is a key parameter for an application in LLE, it has also been assessed for two PLs by evaluating their behavior when contacted with water. Finally, preliminary extraction tests on three rare earth elements (neodymium, praseodymium, dysprosium) generally found in permanent magnets were conducted on PLs after their functionalization with a specific chelating ligand.

## 2 Results and Discussion

### 2.1 Synthesis and Characterization of the Hollow Silica Particles

**Figure 2:**
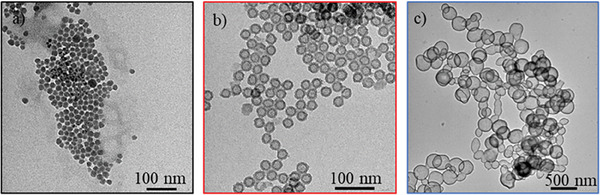
TEM images of a) SiNPs (x80k) b) sHSPs (x80k), and c) bHSPs (x10k).

The basic building blocks of the PLs are the HSPs, whose size, cavity diameter, and shell porosity can be tuned by using different synthesis methods. In this study, three kinds of silica cores were investigated. Small hollow particles, sHSPs were synthesized with the use of the oily 1,3,5‐tetramethylbenzene (TMB) and the amphiphilic F127 as templating agents.^[^ ^14^ ^]^ Calcination of the resulting material provided the expected sHSPs in quantitative yield. Big hollow particles, bHSPs were obtained by following a procedure derived from the Stöber method^[^ ^33^ ^]^ with an additional incubation step in water leading to the formation of the hollow cavities of bHSPs (Figure 1a). Besides, commercially available SiNPs were used as a non‐porous reference. Transmission electron microscopy (TEM) imaging allowed to observe the cavity of the HSPs and to distinguish them from the SiNPs that showed no contrast difference between their core and their surface (**Figure** **2**).

**Figure 3:**
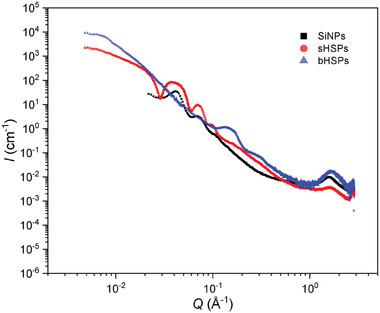
Comparison of the structural organization of SiNPs (black squares), sHSPs (red circles), and bHSPs (blue triangles) powders by SAXS‐USAXS.

It can be noticed that some of the bHSPs present a non‐perfect spherical shape. This can be assigned to the use of a soft template for such big particles.^[^ ^27^ ^]^ The internal and external diameters could be extracted from the images. SiNPs have a diameter of 17.4 ± 2.8 nm, sHSPs are in the same size range with an external diameter of 25.4 ± 1.4 nm, and an internal diameter of 14.8 ± 1.3 nm, while bHSPs are one order of magnitude bigger with mean external and internal diameters respectively of 236.0 ± 64.4 nm and 203.6 ± 58.8 nm respectively (Figure S2, Supporting Information). These results are gathered in **Table** **1** and Table S1 (Supporting Information). TEM results were correlated with those deduced from SAXS and USAXS (**Figure** **3**). The three powders provide an increase in scattering intensity at low angles, which is characteristic of nanospheres. Oscillations were fitted with SasView software for SiNPs and sHSPs. They correspond to monodisperse nanospheres and nanoshells respectively, with diameters consistent with the ones obtained from TEM images (Tables S1,S2 and Figure S3, Supporting Information). The less pronounced oscillations observable for SiNPs are consistent with a higher polydispersity for these particles. bHSPs are found to be too big and polydisperse to provide any proper fit in this *Q* range.

**Table 1:** Relationship between the size, packing density and porous properties of the powders for the three silica cores used to synthesize hybrid liquids.

	External diameter (TEM) [nm]	Internal diameter (TEM) [nm]	Packing density [g cm^−3^]	S_BET_ [m^2^ g^−1^]	Porous volume^b)^ [cm^3^ g^−1^]	Pores size^c)^ [nm]
SiNPs	17.4 ± 2.8	–	1.02 ± 0.06	175	0.42	–
sHSPs	25.4 ± 1.4	14.8 ± 1.3	0.56 ± 0.06	691^a)^	0.66	1.4
bHSPs	236.0 ± 64.4	203.6 ± 58.8	0.12 ± 0.06	981	0.78	1.5/3.7

^a)^ The presence of a high content of microporosity in sHSPs induces an artificial increase in the BET specific surface area;

^b)^ Porous volume recorded at *P*/*P*_0_  =  0.87 to exclude the interparticle void;

^c)^ Pores size in the silica shell for sHSPs and bHSPs, the underlined value corresponds to the most represented.

Additionally to the size of the particles, their density and their porosity are expected to play a role on the dynamics and on the viscosity of the final PL. The packing densities of the three powders were measured by packing a calibrated mass of the materials in a graduated container and measuring their volume (Table 1). SiNPs are the densest particles (1.02 ± 0.06) because of their smallest size and insignificant porosity. In the same manner, sHSPs are denser than bHSPs (0.56 ± 0.06 and 0.12 ± 0.06 respectively) because they are smaller and less porous.

**Figure 4:**
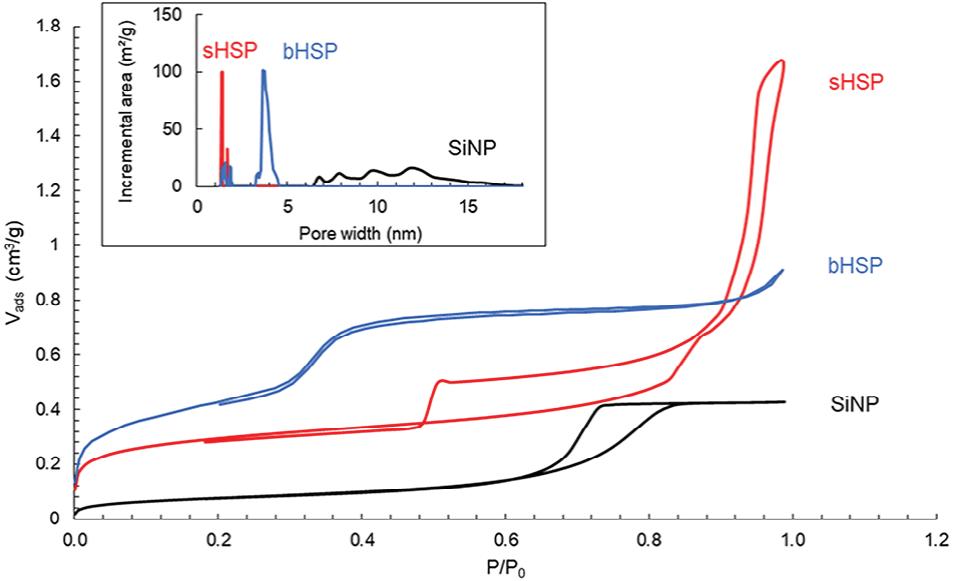
Adsorption/desorption isotherms of N_2_ sorption at 77K in SiNPs (black), sHSPs (red), and bHSPs (blue). Insert: pore size distribution expressed by surface area (DFT).

To correlate the density with the porosity of the spheres, N_2_ sorption measurements were also performed (**Figure** **4**) and the main characteristics are gathered in Table 1. For the SiNPs, a Type IV isotherm (named according to the IUPAC classification) was obtained, with a low volume of adsorbed gas (0.42 cm^3^ g^−1^), suggesting the presence of a few mesopores. Similarly, sHSPs displayed a Type I + Type IV hybrid isotherm, with an H1 hysteresis at relative pressures between 0.5 and 0.87, revealing a large distribution of mesopores (the cavities) and the presence of a microporosity in the shell. The porous volume is estimated at 0.66 cm^3^ g^−1^ (see details in Section S2, Supporting Information), and the diameter of the pores is 1.4 nm based on density functional theory (DFT) calculations (Figure 4, insert). The signal of bHSPs approaches a hybrid Type II + Type IV curve typical of a macroporous solid containing micro‐ and mesopores. In this case, the cavity inside the particle is considered as a macropore because its diameter overcomes 50 nm. Moreover, the presence of two steps (for relative pressures ≈0.005 and 0.3) instead of one indicates two different populations of micro‐ or mesopores in the shell. The porous volume is 0.78 cm^3^ g^−1^ (see Section S2, Supporting Information) and this high porosity is consistent with the lowest density discussed previously. The distribution obtained by DFT confirms that the major part of the pores has a diameter of 3.7 nm and the minor part has a diameter of 1.5 nm. The presence of mesopores is encouraging for application in LLE.

### 2.2 Synthesis and Characterization of the Porous Liquids

#### 2.2.1 Synthesis of Porous Liquids

The PLs synthesis involved the modification of silica spheres with surrounding amphiphilic brushes whose chain mobility, ionic interaction strength, and hydrophilic/lipophilic balance (HLB) were expected as some of the main key parameters ruling the properties of the modified materials. This modification consisted first in the covalent grafting of a sulfonated organosilicon, namely the 3‐(trihydroxysilyl)−1‐propane sulfonic acid (SIT), onto the surface of the silica particles achieved in heated lightly acidic (adjustment at pH 5–6 with NaOH 1 ) aqueous solutions (Figure 1).^[^ ^36^ ^]^ It was then followed by the protonation of the tethered sulfonate groups by using a cation‐exchanger column. Finally, the recovered high sulfonic acid‐containing material was allowed to establish an ionic interaction with stoichiometric amount (neutralization monitored by pH‐meter) of a fatty ethoxylated amine (EthA) carrying two PEG arms containing a tunable number of ethylene oxide units (n and m with n+m = 5, 11, 22, 50, 92; labeled EthA‐5 to EthA‐92). These amines were all commercially available, except for EthA‐92 that was synthesized in the laboratory according to the procedure described by Atta et al.^[^ ^37^ ^]^ by ethoxylation of the stearylamine with the corresponding PEG (see section S1, Supporting Information). All modified materials were distinguished according to the nature of the silica sphere precursor and the n+m value of the associated EthA (e.g., PL‐sHSPs‐22 was made with sHSPs and EthA‐22). For liquids based on SiNPs, they were labeled PL‐SiNPs even though these materials are non‐porous. Their physicochemical properties have been summarized in **Table** **2**.

**Table 2:** Physical state of PLs at room temperature, along with their melting temperature (*T*_m_) in °C and the viscosity (*η*) in Pa.s at 50 °C and $\dot{\gamma }$ = 10 s^−1^ (italic). The picture shows all the PL‐sHSPs at room temperature.

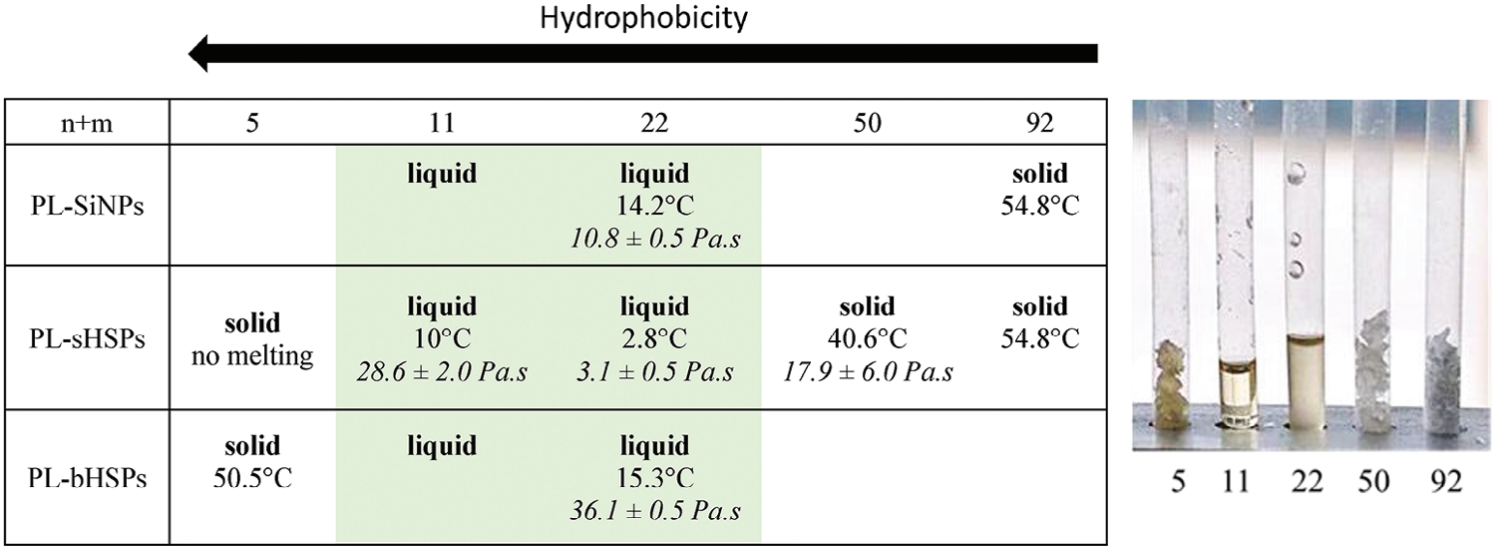

#### 2.2.2 Size and Shape Analysis of PLs

**Figure 5:**
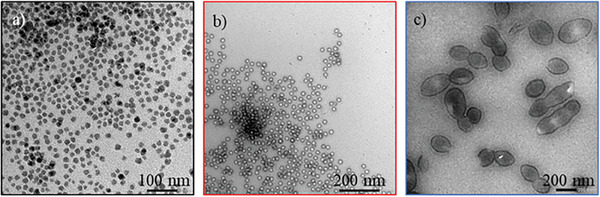
TEM images of PLs a) PL‐SiNPs‐11 (x60k), b) PL‐sHSPs‐11 (x40k), and c) PL‐bHSPs‐11 (x20k).

In **Figure** **5**, TEM images show that the particle's shapes and sizes are not altered by the chemical modification. Additionally, the particles appear less aggregated on the copper grid than in Figure 2, suggesting the presence of the organic molecules between the particles. However, the PEG length seemed to have only little impact on the distance between particles (Figure S4 and Table S4, Supporting Information) and was ≈30 nm in all cases.

Structural analyses of PLs with SAXS and USAXS measurements (Figure S5, Supporting Information) corroborated these results. The remaining oscillations are characteristic of the silica cores integrity. A structural peak is moreover present for all the PLs, corresponding to a correlation distance of 3.9 to 4.0 nm (except for PL‐sHSPs‐92 in Figure S5b). This peak reflects a structural contribution of the amorphous amine since it is also present in the signals of the pristine amines in Figure S5d (except for EthA‐50 and EthA‐92). As observed previously,^[^ ^38^ ^]^ this peak was attributed a nanostructuration of the PEG chains induced by water.

#### 2.2.3 Assessment of the Grafting Efficiency

**Figure 6:**
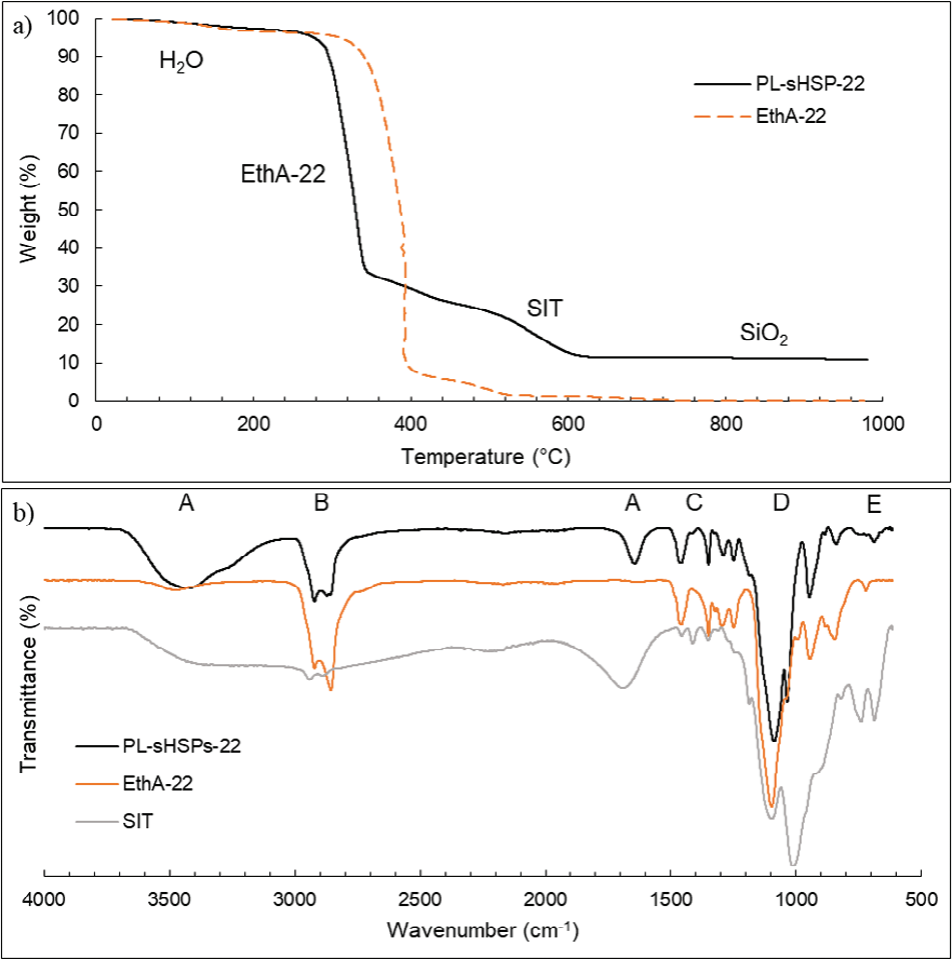
Grafting efficiency of sHSPs in PL‐sHSPs‐22 proved by a) thermal decomposition of PL‐sHSPs‐22 and EthA‐22, b) FTIR spectra of PL‐sHSPs‐22, EthA‐22, and SIT.

The grafting efficiency was primarily evaluated by TGA and FTIR spectroscopy. **Figure** **6**a shows the thermal decomposition of pure EthA‐22 and PL‐sHSPs‐22 under air, at a rate of 5 °C min^−1^.

A large fall in weight (68%) was observed at 325 °C, which is attributed to the deterioration of the canopy. Also, a small percentage of the weight loss (≈6%) was visible at ≈380 °C, which may be due to a molecular weight dispersion of the commercial EthA. Indeed, this dispersion was also visible in the decomposition of the pure amine. Furthermore, the weight loss in the signal of EthA‐22 occurred at higher temperatures, indicating that the presence of silica nanoparticles lowers its thermal stability.^[^ ^39^ ^]^ A second major weight loss at 550 °C (12.4%) was caused by the deterioration of the SIT corona, and the residual weight percentage finally gave the silica content of the PL (10.9% in this case). In addition, at temperatures of ≈100 °C, a small weight loss of 2.6% was revealed, corresponding to residual water contained in the PL. Repetitions of the same measurement lead to water contents varying between 1% and 5%, suggesting that the material is highly hygroscopic. The measurements were performed for all the PLs (Figure S6, Supporting Information), and two histograms are represented in Figure S7 (Supporting Information) to summarize these results (Figure S7a considers the impact of the chains lengths whereas Figure S7b shows the impact of the core nature). When the amine length was varied from n+m  =  5 to 92 (Figures S6a,S7a), the weight content corresponding to the canopy was consistently increased. Details on the molar ratios of Amine/SiO_2_ and Amine/SIT are provided in Tables S5,S6 (Supporting Information).

FTIR spectroscopy confirmed the efficient grafting of the organosilicon and of the polyethoxylated canopy (Figure 6b). Two small absorption bands at 1413 cm^−1^ (C) and 687 cm^−1^ (E) appear after the addition of the SIT onto the silica spheres, which are respectively assigned to the S = O stretching of the sulfonate and to the vibration of the CH_2_ groups of the propyl chain. Two other peaks at 2918 and 2857 cm^−1^ (B) correspond to the stretching of CH_2_ groups and an increase in intensity of these peaks was observed after the addition of the amine due to its long alkyl chain. The very intense band at 1066 cm^−1^ (D) includes both the Si‐O‐Si and the CH_2_‐CH_2_‐O stretching.^[^ ^23^, ^40^ ^]^ The two broad bands at 3433 cm^−1^ and 1645 cm^−1^ (A) are reported to be characteristic of OH bonds in water molecules.^[^ ^23^, ^41^ ^]^ In Figure S8 (Supporting Information), the FTIR signals of all the PL‐sHSPs normalized according to the peak (E) were consistent with an increase in the number of ethoxy groups when varying the amines from 5 to 92.

#### 2.2.4 Physical Properties of Porous Liquids

PLs were further characterized to investigate their macroscopic properties, and how they are influenced by the nature of the silica core and of the canopy. For comparison, it is worthy to note that pure amines EthA‐50 and EthA‐92 are in a solid state whereas the three low molecular weight amines (EthA‐5, EthA‐11, and EthA‐22) are in a liquid state at room temperature. Table 2 gathers the physical states of PLs obtained at room temperature while varying the number of ethoxylated groups of the canopy from 5 to 92, onto three types of silica spheres: SiNPs, sHSPs, and bHSPs.

Among all the PLs based on sHSPs, only PL‐sHSPs‐11, and PL‐sHSPs‐22 display a liquid behavior at room temperature. PL‐sHSPs‐5 becomes solid because of a too‐high ratio of silica content over organic molecules. On the other hand, PL‐sHSPs‐50 and PL‐sHSPs‐92 are also solid at room temperature. This behavior is assumed to be caused by crystalline domains in longer ethoxylated chains with hydrogen bonding occurring and leading to less mobility.^[^ ^24^ ^]^ Additionally, a longer ethoxylated chain is more likely to interact with a neighboring silica particle, creating a network‐like structure.^[^ ^42^ ^]^ DSC measurements allowed to compare the melting temperatures of all the PLs (Table 2; Figure S9, Supporting Information). They are consistent with the previous observations.

Furthermore, the viscosities of the PLs were measured at different temperatures, and Figures S10,S11 (Supporting Information) show the results for PL‐sHSPs‐22. Note that condensation occurred at 15 °C, leading to a significant decrease in viscosity, therefore the corresponding value has not been reported in Figure S10. At 20 °C, the viscosity of PL‐sHSPs‐22 is very high (10^4^ Pa.s), but it exponentially decreases when increasing the temperature: at 50 °C, the viscosity is ≈10^0^ Pa.s. This value is comparable to the ones reported in literature for similar PLs.^[^ ^43^, ^44^ ^]^

The impact of the PEG length of the EthAs on the viscosity was studied at 50 °C. The viscosity of the PLs‐sHSPs at a flow shear rate ($\dot{\gamma }$) of 10 s^−1^ are reported in Table 2. A minimum of viscosity was found for PL‐sHSPs‐22, in accordance with the physical states and the melting points that were discussed previously. However, the viscosity of PL‐sHSPs‐92 was measured at 57 °C, just above its melting point (55 °C), and it happened to be lower than other PLs, with a value of 2.0 ± 0.5 Pa.s. This result suggests that at high molecular weights, PEGylated amines are playing a role of plasticizer and decrease the viscosity, even if the melting point remains relatively high.

The influence of the silica core was also studied by measuring the viscosity of PL‐SiNPs‐22, PL‐sHSPs‐22 and PL‐bHSPs‐22 at 50 °C and 10 s^−1^ (Table 2). The lowest viscosity was obtained for PL‐sHSPs‐22 (3.1 ± 0.5 Pa.s) because of i) the small size of sHSPs with respect to bHSPs, and ii) the small density of sHSPs with respect to SiNPs. An intermediate viscosity of 10.8 ± 0.5 Pa.s was found for PL‐SiNPs‐22. By contrast, PL‐bHSPs‐22 displayed a higher viscosity (36.1 ± 0.5 Pa.s), suggesting that the large diameter of bHSPs contributes to a decrease in molecular mobility, even though the density of these spheres is very low (Table 1). However, it is worthy to note that when it comes to lightest amines, the trend different. In fact, a liquid behavior was observed for PL‐bHSPs‐5 above 50 °C, while PL‐sHSPs‐5 was a solid at any temperature. Thus, in this latter case, the density of the spheres has a more significant impact than their diameter. In Conclusion, the obtained results illustrate a balance between the particles sizes and densities in the contribution to the viscosity of the final PL, that is intrinsically linked to the size of the organic canopy.

#### 2.2.5 Hydrophobicity and Stability in Water

By varying the number of ethoxylated units in the lateral PEG chains of the amine in the canopy of PLs, it was possible to obtain either hydrophilic or hydrophobic materials.

**Figure 7:**
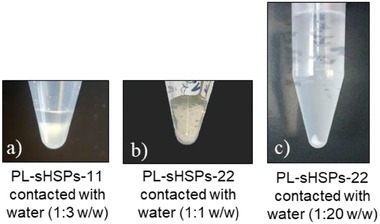
Impact of contacting water with PL‐sHSPs‐11 and PL‐sHSPs‐22.

A simple test was first conducted by mixing a small quantity of PL‐sHSPs‐11 and PL‐sHSPs‐22 with water (1:3 w/w and 1:1 w/w respectively). After centrifugation, PL‐sHSPs‐11 separated well from the aqueous phase (**Figure** **7**a) whereas PL‐sHSPs‐22 and water were forming one viscous phase (Figure 7b). When adding more water (1:20 w/w, Figure 7c), PL‐sHSPs‐22 was totally solubilized in the aqueous phase and the centrifugation just allowed to observe a small fraction of silica in the bottom of the tube. Therefore, it can be assumed that the amine was solubilized in water, leading to the deterioration of the canopy of the PL in this latter case.

**Figure 8:**
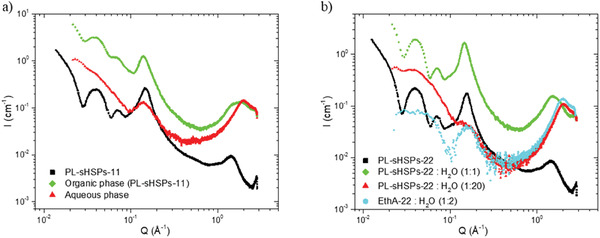
Effect of the water on the structure of a) PL‐sHSPs‐11 measured by SAXS. Black: PL‐sHSPs‐11 before contact, green: PL‐sHSPs‐11 after contact, and red: the aqueous phase of the contact test, b) PL‐sHSPs‐22. Black: pure PL‐sHSPs‐22, green: PL‐sHSPs‐22 and a small amount of water (1:1 w/w), red: PL‐sHSPs‐22 and a huge amount of water (1:20 w/w), cyan: EthA‐22 solubilized in water (1:2 w/w).

In order to confirm these results, SAXS measurements have been achieved on the different phases of the samples (**Figure** **8**). In Figure 8a concerning PL‐sHSPs‐11, the oscillations and the structural peak at 0.16 Å^−1^ characteristic of the PLs is maintained after contact with water, suggesting that the structure of the PL was not altered by the water. However, some water entered the PL, as indicated by the large “solvent” peak ≈1.5 Å^−1^ that indicates the presence of both silica and water. In the aqueous phase, the oscillations disappeared but the increase in intensity at small angles can be attributed to the presence of silica shells and organic molecules. Hence, a small amount of PL is expected to be solubilized in water. In the case of PL‐sHSPs‐22 (Figure 8b), a small amount of water does not significantly change the structure of the PL whereas a higher dilution leads to a signal really similar to the amine EthA‐22 in water (cyan curve). This corroborates the previous observation of a precipitate in Figure 7c: the solid corresponds to the sHSPs grafted with the sulfonated organosilicon derivative while the amine EthA‐22 is solubilized in water. These results suggest that there is a threshold in the number of ethoxylated units that can be present without making the PL hydrophilic.

TGA and total organic carbon (TOC) analyses were also performed to quantitatively support these results. In the case of PL‐sHSPs‐11 contacted with water, the organic phase contained 51% of water, but it dried spontaneously at room temperature in few hours. It is assumed that this water was trapped inside the silica particle and the first hydrophilic corona. After drying, the TGA signal totally overlapped the one of PL‐sHSPs‐11 before contact, confirming the SAXS data concerning the stability of this PL against water (Figure S12a, Supporting Information). Furthermore, 10% of the PL‐sHSPs‐11 was lost in the aqueous phase, according to the TOC analysis. This small fraction confirms the hydrophobicity of the PL conferred by the amine EthA‐11. In the case of PL‐sHSPs‐22, TGA showed that after aqueous contact 6.4% of organic molecules were present in water, along with 0.7% of silica (Figure S12b, Supporting Information). These values lead to a 100% of the initial weight of PL‐sHSPs‐22 that had been dissolved in water (except for a negligible weight of silica that has precipitated).

At this point, it is interesting to note that because EthAs with n+m up to 50 were industrial amphiphilic compounds derived from tallow mixtures, the length of the alkyl chain could not be accurate and lay between 16 and 18 carbon atoms. In the case of EthA‐92 that was synthesized in the laboratory, the number of carbon atoms was set to 18 as the starting material was the octadecylamine. In all cases, these carbon chains were considered to provide a sufficient hydrophobic character to the overall material. Therefore, this study concerned only the influence of PEGs length, and no peculiar attention was paid to that of the alkyl tail.

### 2.3 Functionalization and Application in Rare Earth Extraction

To apply PLs in LLE, not only have they to be hydrophobic and contain large pores, but a functionalization is also expected to ensure an enhanced and specific extraction of the targeted metal. In this study, functionalization of the sHSPs by co‐condensation of tetramethyl orthosilicate (TMOS) with silanized ethylenediaminetetraacetic acid (Si‐EDTA) was performed at 16:1 TMOS:Si‐EDTA molar ratio, and rather than calcination, a further step of washing with EtOH and HCl (pH 1) for 24 h was implemented. Preliminary tests of LLE on three rare earth elements (neodymium, praseodymium, dysprosium) were performed. To simulate a magnet lixiviate, they were dissolved in nitric acid at pH 2 at initial concentrations of 500, 68, and 50 ppm for Nd, Pr, and Dy respectively. This solution was contacted with either PL‐sHSPs‐11 or PL‐sHSPs‐SiEDTA‐11 during 24 h at room temperature. The metal concentrations in the aqueous phases were measured by Inductively Coupled Plasma (ICP) analysis and the resulting extraction yields and extraction capacities are given in **Table** **3**. The PL‐sHSPs‐11 with no functional groups did not show any extraction of rare earth elements, whereas PL‐sHSPs‐SiEDTA‐11 displayed measurable extraction yields and significant loading capacities (*Q*_e_) for the three metals. This proof of concept therefore shows that the presence of functional groups is necessary to perform efficient LLE with such PLs.

**Table 3:** Extraction yield and capacity of Nd, Pr, and Dy by PL‐sHSPs‐11 and PL‐sHSPs‐SiEDTA‐11.

	PL‐sHSPs‐11		PL‐sHSPs‐SiEDTA‐11	
	*E*_%_ [%]	*Q*_e_ [mmol g^−1^]	*E*_%_ [%]	*Q*_e_ [µmol g^−1^]
Nd	Traces	–	2.3	71.9
Pr	Traces	–	6.0	26.7
Dy	Traces	–	4.3	11.5

## 3 Conclusion

A range of new silica‐based porous liquids (PLs) based on modified silica spheres reversibly surrounded by an amphiphilic canopy has been developed by successfully varying the nature of both silica cores and canopies. For the sake of comparison, small non‐porous silica nanoparticles (SiNPs) and porous hollow silica particles (HSPs) of two different sizes were first designed as starting silica cores before their surface modification with sulfonic acid groups. Acido‐basic reaction between these functional groups and an ethoxylated fatty amine (EthA) lead to the formation of the canopy. Liquefying properties and hydrophobicity were also both tuned for the first time according to the number of ethoxylated units (n+m) born by the EthA, which was varied from 5 to 92. Only six synthesized materials were observed to display a liquid behavior at room temperature. Considering the effect of the silica particle nature, it was evidenced that the density and the diameter of the silica spheres both played a role on the physical properties of the final PLs. Comparing SiNPs and sHSPs, the lower density of sHSPs implied a decrease in viscosity of the final PL. Besides, PLs obtained with these latter silica cores were less viscous than the one obtained by using bHSPs, even though they are much less dense. Regarding the effect of the organic grafting, it has been shown that the physical state of the final material also strongly depends on the number of ethylene oxide units in the EthA that forms the canopy. Hence, intermediate‐range molecular weight amines (n+m = 11 and 22) lead to liquids at room temperature, whereas high molecular weight amines lead to solids that melted at low temperature (40–55 °C). The lightest amine did not allow to obtain a liquid at any temperature when grafted on sHSPs. In addition, using EthA‐11 led to hydrophobic PLs whereas EthA‐22 made the PLs hydrophilic. In order to test the hydrophobicity of the obtained PLs, PL‐sHSPs‐11, and PL‐sHSPs‐22 were contacted to pure water. A solubilization in water was observed for the less hydrophobic PL‐sHSPs‐22, while a clear phase separation could be obtained with the more hydrophobic PL‐sHSPs‐11. This unprecedented result is an essential prerequisite for considering applying PLs to liquid‐liquid extraction (LLE). Preliminary LLE tests have been performed on three rare earth elements (Nd, Pr, and Dy). It was shown that metal extraction is possible with such hydrophobic PLs and that it requires their previous functionalization with a chelating ligand (EDTA). Further studies will include an optimization of the ratio chelating ligand:silica as well as the evaluation of other chelating groups like diglycolamide to enhance the intra‐lanthanide selectivity of extraction. Finally, since the use of a short organosilicon could lead to a blockage of the microporosity in the silica shell, therefore decreasing the accessibility to the empty cavities, optimization of the synthesis should include the use of a bigger organosilicon, or an efficient removal of the template after the organosilicon grafting.

## 4 Experimental Section

### Materials

Ludox HS40, tetramethyl orthosilicate (TMOS), (3‐mercaptopropyl)trimethoxysilane (MPTMS), potassium sulfate (K_2_SO_4_), tetraethyl orthosilicate (TEOS), cetyltrimethylammonium bromide (CTAB), PluronicF127, and 1,3,5‐trimethylbenzene (TMB) were purchased from Sigma–Aldrich. Aqueous ammonia (NH_4_OH, 25%) was purchased from Fisher. 3‐(trihydroxysilyl)−1‐propane sulfonic acid (SIT, 30–35%) was purchased from Gelest. Ethanol (EtOH) and sodium hydroxide (NaOH) were purchased from Carlo Erba. Silanized ethylenediaminetetraacetic acid (Si‐EDTA, 45%) was purchased from ABCR. Ethoxylated amines (EthAs) came from three different sources: Rokamin SR5 (EthA‐5), Rokamin SR11 (EthA‐11), and Rokamin SR22 (EthA‐22) were supplied by PCC Group, Ethox SAM50 (EthA‐50) was supplied by Ethox Chemicals, and EthA‐92 was synthesized according to method reported by Atta et al.^[^ ^37^ ^]^ The detailed procedure as well as NMR spectra of EthA‐22, EthA‐50, and EthA‐92 are included in the Supportin Information (see Section S1, Supporting Information). Deionized water with a resistivity of 18.2 MΩ.cm and a TOC value below 5 ppb was provided by a Milli‐Q direct 8 water purifier device (Merck, France).

### Synthesis of Hollow Silica Particles Syntheses

sHSPs were synthesized according to the method described by Zhang et al.^[^ ^14^ ^]^ as follows: 8.33 g (0.66 mmol) of F127, 8.33 g (69.3 mmol) of TMB, and 7.25 g (41.6 mmol) of K_2_SO_4_ were mixed together with 500 mL of water in a 1L‐round bottom flask maintained at 13.5 °C, and stirred during 4 h. Then, 20.25 g (133.0 mmol) of TMOS and 6.5 g (33.1 mmol) of MPTMS were added and the mixture was let at 13.5 °C during 24 h then aged at 100 °C under stirring during 24 h. The product was then filtered on a 5–13 µm paper and washed three times with water. It was then calcinated during 10 h at 450 °C and the solid (c.a. 10 g) was grinded before being used.

bHSPs synthesis was inspired from the method of Teng et al.^[^ ^33^ ^]^ that was derived from the Stöber method.^[^ ^45^ ^]^ It consisted in dissolving 0.450 g (1.23 mmol) of CTAB in a solution containing 52.5 mL of EtOH, 150 mL of deionized H_2_O, and 3 mL (40.2 mmol) of a 25% aqueous NH_3_ solution. 3 mL (13.4 mmol) of TEOS was added quickly and the suspension was let under stirring during 24 h at room temperature to make the condensation of TEOS. The product was washed three times with EtOH by centrifugation, before being incubated in 1.2 L of water and heated at 70 °C during 20 h. The bHSPs were washed three times with EtOH. To remove the CTAB template, 360 mL of an EtOH:HCl 10^−3^ n mixture was heated at 60 °C, and the spheres were suspended overnight in this medium. The spheres were recovered after centrifugation and they were then washed three times in EtOH prior to a further extraction performed in a EtOH: HCl 10^−2^ n mixture heated at 60 °C during 3 h. The final product (c.a. 400 mg) was obtained after washing three times in EtOH and drying under vacuum.

### Porous Liquids Syntheses of PL‐SiNPs Synthesis

PL‐SiNPs synthesis was inspired by the work of Rodriguez et al.^[^ ^36^, ^46^ ^]^ concerning Nanoscale Ionic Materials (NIMs): 2.860 g (19.0 mmol) of Ludox HS40 solution were diluted in 25 mL of deionized water (from Milli‐Q). 6.4 g (10.1 mmol) of SIT solution were diluted in 25 mL of deionized water, in a 100 mL‐round bottom flask. The silica suspension was added dropwise to the SIT solution under vigorous stirring. The pH was adjusted at pH 5–6 with NaOH 1 m, then the solution was heated at 70 °C to start the hydrolysis and it was left at 70 °C for 24 h. It was then dialyzed with a 6000–8000 kDa membrane to remove the excess of NaOH and unreacted SIT prior to elution through an ion‐exchange column (Dowex 50W‐X8) to protonate the sulfonate salts. The Total Organic Carbon (TOC) analysis of the dialysis external water baths allowed determining the amount of unreacted SIT (≈76%). Hence, 2.4 mmol of SIT were grafted upon 1.14 g (19.0 mmol) of silica spheres. Finally, a v:v 100 times diluted aqueous solution of EthA was prepared (the dilution was only ten times for EthA‐92) and added dropwise to the solution of grafted nanospheres. The addition, which was followed by pH measurement, could be stopped just after the equivalence point. It was useful to highlight that this equivalence point led to only 0.09 mmol of SIT, meaning that either the protonation in the column was not total, or the column retained some product inside. The product was dried under vacuum at 35 °C before yielding the expected material, namely PL‐SiNPs‐n+m, where n+m is the global number of the ethylene oxide units in the EthA (e.g., PL‐SiNPs‐22 when n+m  =  22). As an example, the overall yield of PL‐SiNPs‐11 was 3%, mainly due to a poor dispersion of the silica spheres in water (see details in Table S3, Supporting Information). This point might be improved by an ultrasonic‐assisted reaction between the spheres and the SIT.

### PL‐sHSPs Synthesis

2 g of sHSPs was dispersed in 325 mL of deionized water and mixed at 15 000 rpm with an Ultra‐turrax rotor during 5′. After 1 min of sonication (30 W), the dispersion was added dropwise to a solution containing 14.3 g (22.6 mmol) of SIT solution and 325 mL of deionized water under vigorous stirring. The following steps (pH adjustment, dialysis, protonation of tethered sulfonate groups, and subsequent neutralization by the EthA) were performed according to similar procedures to those described for PL‐SiNPs but with two slight adaptations. First, the solution recovered after the reaction with the SIT was concentrated by evaporation under reduced pressure to 100 mL before pouring it into dialysis tubes. Second, the solution recovered after the dialysis was centrifuged at 4000 g for 10′ in order to eliminate some precipitated aggregates. PL‐sHSPs‐11 was obtained with an overall yield of 4% (Table S3, Supporting Information).

### PL‐bHSPs Synthesis

PL‐bHSPs synthesis was achieved following a similar procedure to that described above. 300 mg of bHSPs were dispersed in 50 mL of deionized water and the resulting solution was then added dropwise to a solution containing 3 g (4.75 mmol) of SIT solution and 50 mL of deionized water under vigorous stirring. Subsequent treatments and procedures were applied using the same method as described for PL‐sHSPs. PL‐bHSPs‐11 was obtained in 1% overall yield (Table S3, Supporting Information).

### Synthesis of a Porous Liquid Functionalized with EDTA (PL‐sHSPs‐SiEDTA‐11)

PL‐sHSPs‐SiEDTA‐11 was synthesized according to a similar method to that described for sHSPs synthesis. F127 (1 g), K_2_SO_4_ (0.72 g), and TMB (1.2 mL) were dissolved in 60 mL of deionized water and left for 4 h at 13.5 °C under stirring. Then, 2.48 g (16.0 mmol) of TMOS and 0.87 g (1.0 mmol) of SiEDTA were added to the solution. After 24 h, the temperature was set to 100 °C for another 24 h. Afterward, the mixture was filtered on 5–13 µm paper and washed three times with water to obtain a wet white gel. The material was dissolved in 50 mL of deionized water and dispersed with an Ultra‐turrax rotor (5′) then sonicated for 1′. A solution of SIT (3.58 g) in 50 mL of deionized water was prepared, and the suspension of silica spheres was added dropwise under vigorous stirring. The pH was adjusted to 5–6 with NaOH 1 m and the mixture was heated for 24 h at 70 °C. After 5 days of dialysis with deionized water (8 batches), the suspension was centrifuged to eliminate non‐grafted spheres. The supernatant was dried and dispersed again in a EtOH/HCl solution (pH 1). The dispersion was heated to reflux under stirring for 24 h to extract the template. After a filtration and three washing with ethanol, the solid was dried and dispersed again in water, before elution through an ion‐exchange column to protonate the sulfonate salts and obtain sHSPs‐SiEDTA@SIT. Finally, it was grafted with EthA‐11 by following the previous procedure and the functionalized PL (PL‐sHSPs‐SiEDTA‐11) was collected after drying under vacuum.

### Characterization Methods

Total organic carbon (TOC) measurements were performed with a Shimadzu TOC‐VCSH analyzer calibrated with potassium phthalate solution.

Pure SiNPs, HSPs, and PLs were imaged by Transmission Electron Microscopy (TEM) on a JEOL (Tokyo, Japan) 1400+ at 100 kV, equipped with a sCMOS JEOL Matataki Flash camera and a LaB6 filament. The solid samples were first dissolved in EtOH before their deposition on a carbon‐coated grid whereas those that were liquid at room temperature were not diluted in solvent (except for PL‐bHSPs‐11 that was dissolved in EtOH to increase the contrast). Sizes were measured with ImageJ software, on a minimum of 200 spheres (core, shell, total) to get a representative panel of the sample.

Structural information about HSPs and PLs was obtained thanks to Small Angle X‐Ray Scattering (SAXS) and Ultra‐Small Angle X‐Ray Scattering (USAXS) measurements. The SAXS device was a home‐built one at the ICSM (Marcoule, France) using a bench built by Xenocs with a molybdenum anode as X‐ray Source (0.71 Å) and a MAR Research 345 detector at 770 mm from the sample. The USAXS device was a homemade instrument located at the SWACS‐Lab platform (CEA, Saclay, France), equipped with a X‐ray source (8 keV) that produced a collimated beam and a flux of 0.8×0.8 mm and 108 photons s^−1^ on the sample respectively. The detector (Dectris Pilatus 200K) was located at 114 cm from the sample and calibration was achieved by using tetradecanol. Samples (powders and liquids) were put inside 2 mm‐thick capillaries. PL‐bHSPs‐11 was too viscous to enter a capillary, therefore a Kapton adhesive tape was used instead. The exposition time was 1800 s except for sHSPs (600 s). Calibration was done using a polyethylene standard. Data treatment was done with PySAXS 3.26 and modeling was performed with SasView 5.0.4.

The specific surface area of HSPs was deduced from N_2_ sorption experiments coupled to a Brunauer‐Emmett‐Teller (BET) analysis. The pore size distribution was computed thanks to the Barrett‐Joyner‐Halenda (BJH) model and the Density Functional Theory (DFT). The device was a Micromeritics TriStar II 3020 (Georgia, USA). HSPs and SiNPs were previously outgassed with a Micromeritics VacProp 061 at 80 °C for 48 h.

Thermogravimetric Analyses (TGA) gave access to the composition of PLs. The measurements were performed on a Mettler Toledo (Greifensee, Swiss) device with the STARe Software V13.00, under air atmosphere, from 25 °C to 1000 °C at a rate of 5 °C min^−1^. The samples (≈10–20 mg) were kept in a 70 µL alumina's crucible.

Differential Scanning Calorimetry (DSC) was used to determine the melting temperature of PLs and EthA. The measurements were conducted on a Mettler Toledo DSC1 located in the University of Montpellier, France, under N_2_ atmosphere. First, the samples were cooled downed to −100 °C at 5 °C min^−1^. After an isotherm lasting 5 min, a heating rate of 4 °C min^−1^ was applied to reach 150 °C. The melting temperature is taken at the melting peak maximum.

Fourier Transform Infrared Spectroscopy (FTIR) was carried out on Perkin Elmer (Wellesley, MA, USA) Spectrum 100 spectrometer in Attenuated Total Reflectance (ATR) transmission mode. A small amount of powder or net liquid was homogeneously dispersed on the beam path. The wavenumber range was from 615 to 4000 cm^−1^. The beam resolution was 4 cm^−1^. Each measurement was repeated four times.

The viscosity of PLs was analyzed at different temperatures on a RM200 CP‐4000 cone‐plate rheometer from Lamy Rheology (Limonest, France), equipped with a MK‐CP2020 cone (angle 2°, diameter 20 mm) and a Peltier plate able to set a temperature between 15 and 60 °C. The gap was set to 50 µm. The curves could be modeled by a Bingham fitting (with an exception for the viscosity at 20  °C that did not follow the same behavior) directly on the Rheomatic‐P V2.1.0.4 software. The values of viscosities obtained with this model were close to the one obtained at 10 s^−1^ flow shear rate for each PL (corresponding to a shear rate at the beginning of the zone of Newtonian behavior).

Hydrophobicity of PL‐sHSPs‐11 and PL‐sHSPs‐22 was determined by contacting a certain amount of PL with water at various water:PL weight ratios, and the phase separation was assessed after, according to the involved PL, 5 to 20 min of centrifugation at 4000 to 15 500 *g*. The TOC analysis of the aqueous phase was used to determine the quantity of organic molecules that were transferred into the aqueous phase.

Metal concentrations in the aqueous phase before and after extraction were analyzed by Inductively Coupled Plasma Optical Emission Spectroscopy (ICP‐OES) on an iCAP 7400 Duo Full MFC from Thermo Scientific (Waltham, Massachusetts). The software was Thermo Scientific Qtegra Intelligent Scientific Data Solution Version 2.10. Samples were first diluted 100 times in HNO3 1%. A calibration line was obtained from standard solutions (1000 mg L^−1^, SCP Science).

### Metal Extraction

An aqueous solution containing 500 ppm of Nd, 68 ppm of Pr, and 50 ppm of Dy in nitric acid at pH 2.2 was prepared. 5.0 mL of this solution was contacted with either 30 mg of PL‐sHSPs‐11 or 50 mg of PL‐sHSPs‐SiEDTA‐11 and it was let under stirring at room temperature for 24 h. The two phases were separated immediately and the aqueous phase was analyzed by ICP. The extraction efficiency *E_%_* was defined as:

(1) $$E_{\%}=\left(1-\frac{C_{\mathrm{MA}}}{C_{\mathrm{Moth}}}\right)\times 100$$

With *C*_*Moth*_ the massic concentration of the metal cation M into the mother solution and *C*_*MA*_ its massic concentration into the aqueous phase after extraction.

The loading capacity *Q*_*e*_ (mmol.g^−1^) was computed according to the following equation:

(2) $$\mathrm{Qe}=\frac{\frac{C_{\mathrm{Moth}}-C_{\mathrm{MA}}}{\mathrm{MW}}\times V_{\mathrm{sol}}}{m_{\mathrm{mat}}}$$

With *MW* the molecular weight of the metal, *m*_*mat*_ the mass of the porous material, and *V*_*sol*_ the volume of the aqueous phase.

## Conflict of Interest

The authors declare no conflict of interest.

## Supporting information

Supporting Information

## Data Availability

The data that support the findings of this study are available in the supplementary material of this article.
