# Supplementary material for: Hydrophobic Porous Liquids with Controlled Cavity Size and Physico‐Chemical Properties
Source: Adv Sci (Weinh). 2023 Nov 30;11(4):2305906. doi: 10.1002/advs.202305906 (PMC10811500; doi:10.1002/advs.202305906)
Supplement: Supplementary file 1 — Supporting Information [file ADVS-11-2305906-s001.pdf]

ADVANCED SCIENCE
Open Access

Supporting Information

for *Adv. Sci.*, DOI 10.1002/advs.202305906

Hydrophobic Porous Liquids with Controlled Cavity Size and Physico-Chemical Properties

Lorianne Ginot, Amal El Bakkouche, Fabrice Giusti, Sandrine Dourdain and Stéphane Pellet-Rostaing*

Supporting Information

Hydrophobic Porous Liquids with Controlled Cavity Size and Physico-Chemical Properties

Lorianne Ginot, Amal El Bakkouche, Fabrice Giusti, Sandrine Dourdain and Stéphane Pellet-Rostaing*

ICSM, Univ Montpellier, CEA, CNRS, ENSCM, 30207 Marcoule, France
E-mail: sandrine.dourdain@cea.fr

S1. Synthesis of EthA-92 and characterization of all EthA

A mixture of 13.5 g (50.1 mmol) of octadecylamine (ODA), 200 g (97.6 mmol) of poly(ethylene glycol) (Mn ~ 2050 g/mol, Sigma-Aldrich) (PEG-2050), 14.2 g (99.3 mmol) of 2,2-dichlorodiethyl ether (DDE), and 7.9 g (197.5 mmol) of NaOH in 270 mL of xylene was refluxed under N_2 atmosphere for 5h. After cooling to room temperature, the solvent was filtered under suction in order to remove the precipitated salt (NaCl) and the filtrate was evaporated under reduced pressure. The residual powder was washed with diethylether (to remove unreacted amine and low molecular weight by-products) and then recrystallized in ethylacetate. Crystals were collected after filtration under suction and subsequent washing with cold ethylacetate and pentane. The product was dried under vacuum for 4h to yield 165g (75%) of the expected compound as a white powder, and it was characterized by NMR (Figure S1).

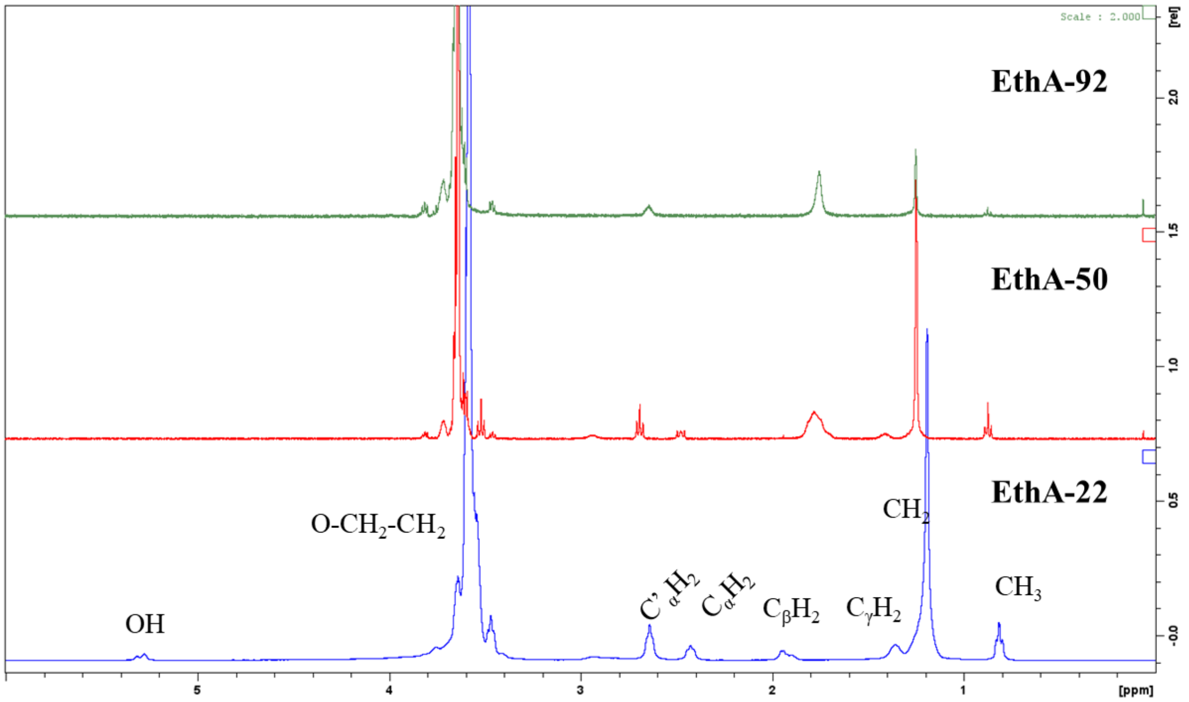

Figure S1. stacking of the ^{1}H NMR spectra of EthA-22 (blue), EthA-50 (red) and EthA-92 (green). Similar spectra can be observed, be it for commercial or synthesized amines.

S2. Characterization of silica core particles by TEM and SAXS experiments

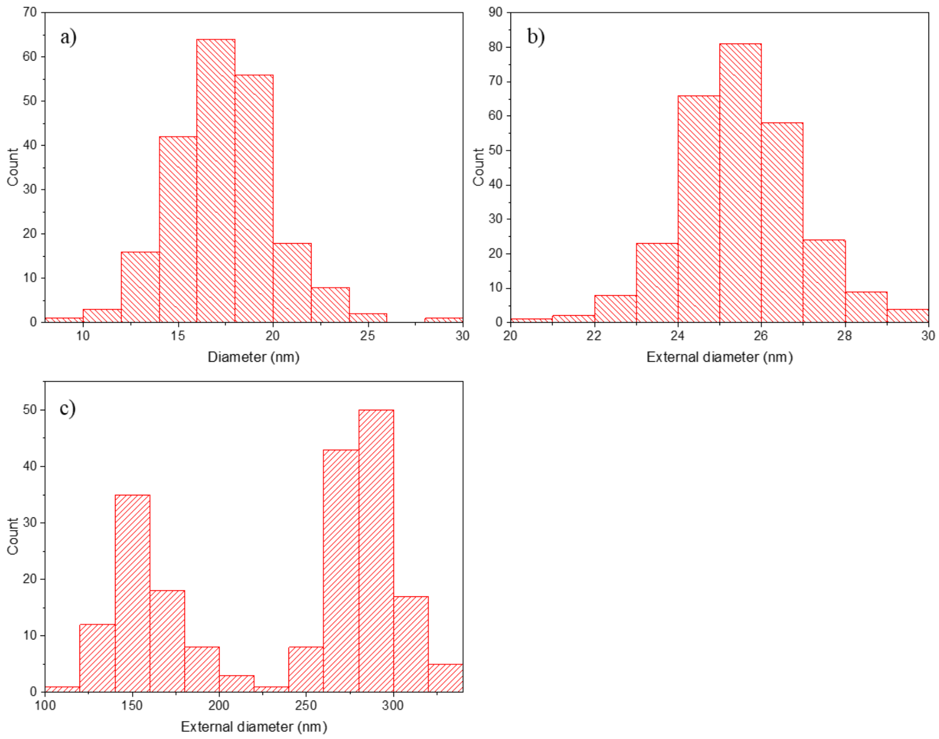

Figure S2. Statistical repartition (# > 200) of external diameters deduced from TEM data analysis of a) SiNPs, b) sHSPs, and c) bHSPs.

For bHSPs, there were two populations of spheres due to the use of a soft template: around 40% had an external diameter of around 149 nm and 60% had an external diameter of around 278 nm in average.

Table S1. Diameters and standard deviations of SiNPs, sHSPs and bHSPs deduced from TEM and SAXS measurements.

	External diameter (TEM) (nm)	Internal diameter (TEM) (nm)	External diameter (SAXS) (nm)	Internal diameter (SAXS) (nm)
SiNPs	17.4 ± 2.8	-	16.2 ± 1.5	-
sHSPs	25.4 ± 1.4	14.8 ± 1.3	24.6 ± 1.5	16.2 ± 1.5
bHSPs	201.6 ± 46.6	184.9 ± 50.3	-	-

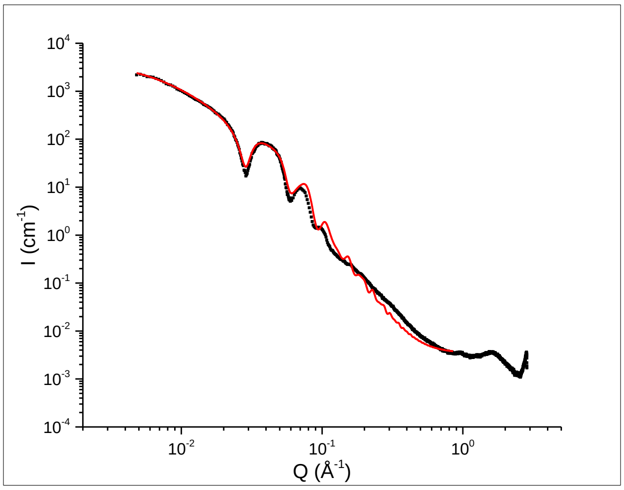

Figure S3. USAXS-SAXS signal of sHSPs (black symbols) and fitting curve (red line) obtained with the software SasView over the range [0.00482;0.853] Å^{-1}. The model used for fitting was a core-shell sphere structure with a sticky hardsphere behavior. The parameters and their value are listed in the Table S1.

Table S2. Parameters for the fit of sHSPs in SasView (see Figure S3).

Internal radius (Å)	81.0
Thickness (Å)	40.1 (silica shell)
Sld core (10^{-6}/Å^2)	0
Sld shell (10^{-6}/Å^2)	18
Sld solv (10^{-6}/Å^2)	0

Volume fraction	0.28
Perturbation	0.05
Stickiness	0.14
Radius polydispersity (ratio)	0.035
Thickness polydispersity (ratio)	0.4

BET analysis. *For sHSPs:* Since the second hysteresis observed in the P/P_0 range between 0.9 and 1 was attributed to the interparticle void, an estimation of the total adsorbed volume of gas inside the particle is 0.66 cm^3/g, taken at $P/P_0 = 0.87$.

For bHSPs: The adsorbed volume of gas at $P/P_0 = 0.87$ is 0.78 cm^3/g in this case (it excludes the interparticle void even though it cannot be totally uncorrelated from the large hollow core of bHSPs).

S3. Characterization of PLs by TEM, SAXS, TGA and DSC experiments

Table S3. Yields of some PLs syntheses at each step.

	SIT grafting (% of spheres)	Colum (protonation and/or retention)	EthA grafting (%)	Overall yield (%)
**PL-SiNPs-11**	100	4	78	3
**PL-sHSPs-11**	7	12	100	1
**PL-bHSPs-11**	24	23	80	4

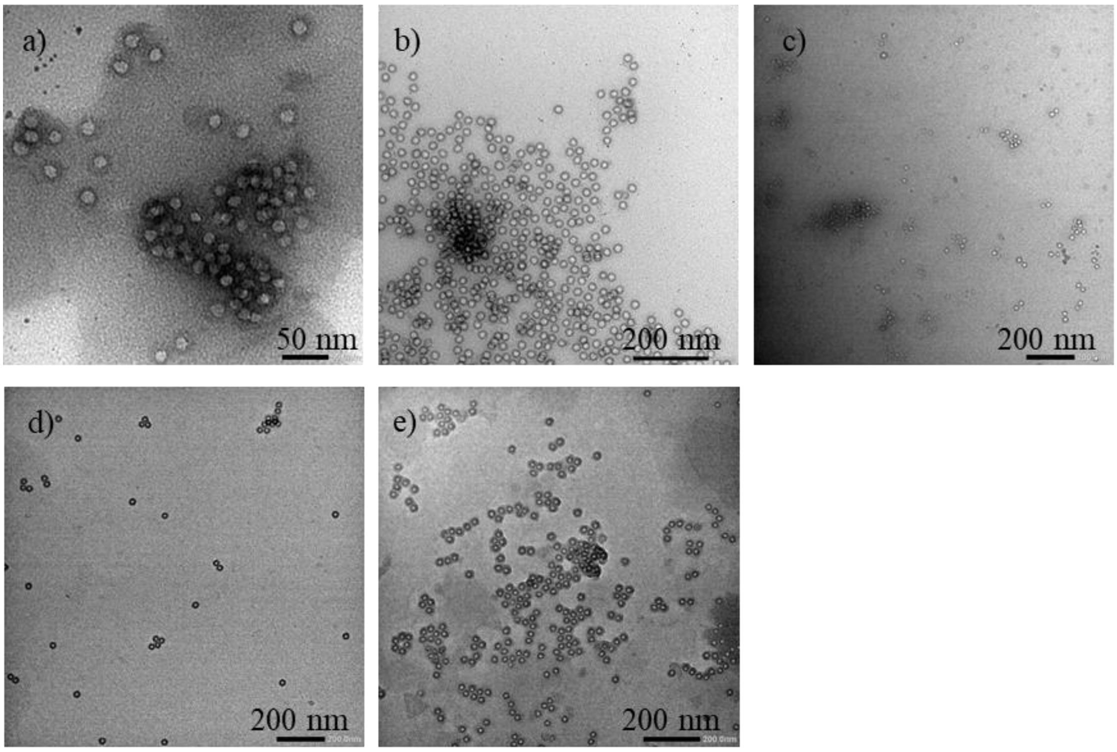

Figure S4. TEM images of PL-sHSPs-5 (x100k), PL-sHSPs-11 (x40k), PL-sHSPs-22 (x25k), PL-sHSPs-50 (x25k) and PL-sHSPs-92 (x30k).

Table S4. Impact of the global PEGs length of the EthA on the inter-particle distance measured from the centers of the particles.

	d_{inter} (nm)	SD (nm)
**PL-sHSPs-5**	28.9	4.0
**PL-sHSPs-11**	28.2	3.4
**PL-sHSPs-22**	31.7	3.1
**PL-sHSPs-50**	26.8	3.3
**PL-sHSPs-92**	27.3	3.7

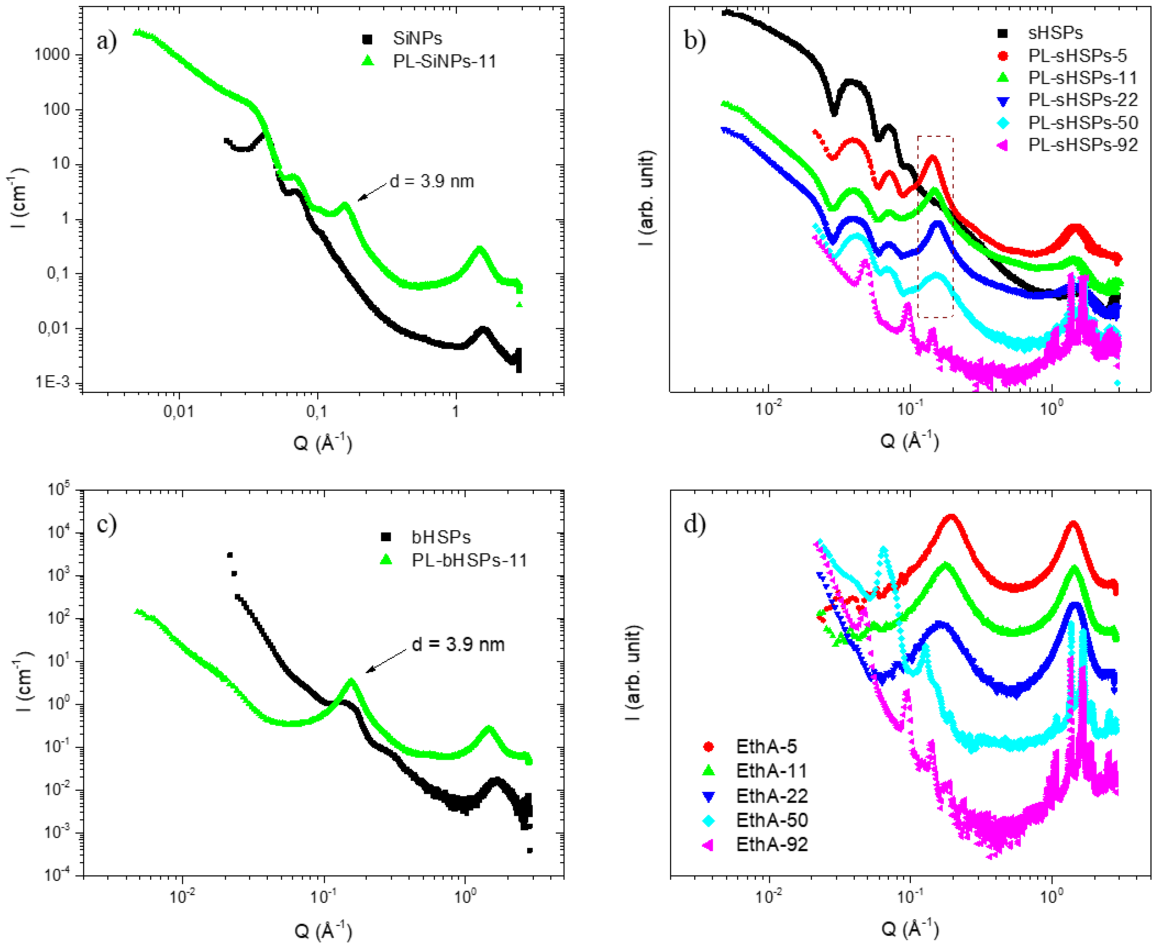

Figure S5. Structural properties of PLs with respect to the properties of the silica spheres and of the free amines. a) SiNPs and PL-SiNPs, b) sHSPs and PL-sHSPs, c) bHSPs and PL-bHSPs, and d) pure amines.

In Figure S5b, the structure of PL-sHSPs-92 does not show the oscillations characteristic of the sHSPs. Instead, regularly spaced structural peaks are visible, that may reflect either a lamellar structure of an onion-like organization at molecular scale. These structural peaks are also visible in EthA-92, and they probably hide the signal of the sHSPs because of the high molecular weight of the amine. In another hand, PL-sHSPs-50 displays a structural peak at 0.16 $Å^{-1}$ like other PL-sHSPs, even though no such peak was visible in the signal of EthA-50. It is interpreted as an intermediate between the highly organized EthA-92 and the poorly organized short amine EthA-22.

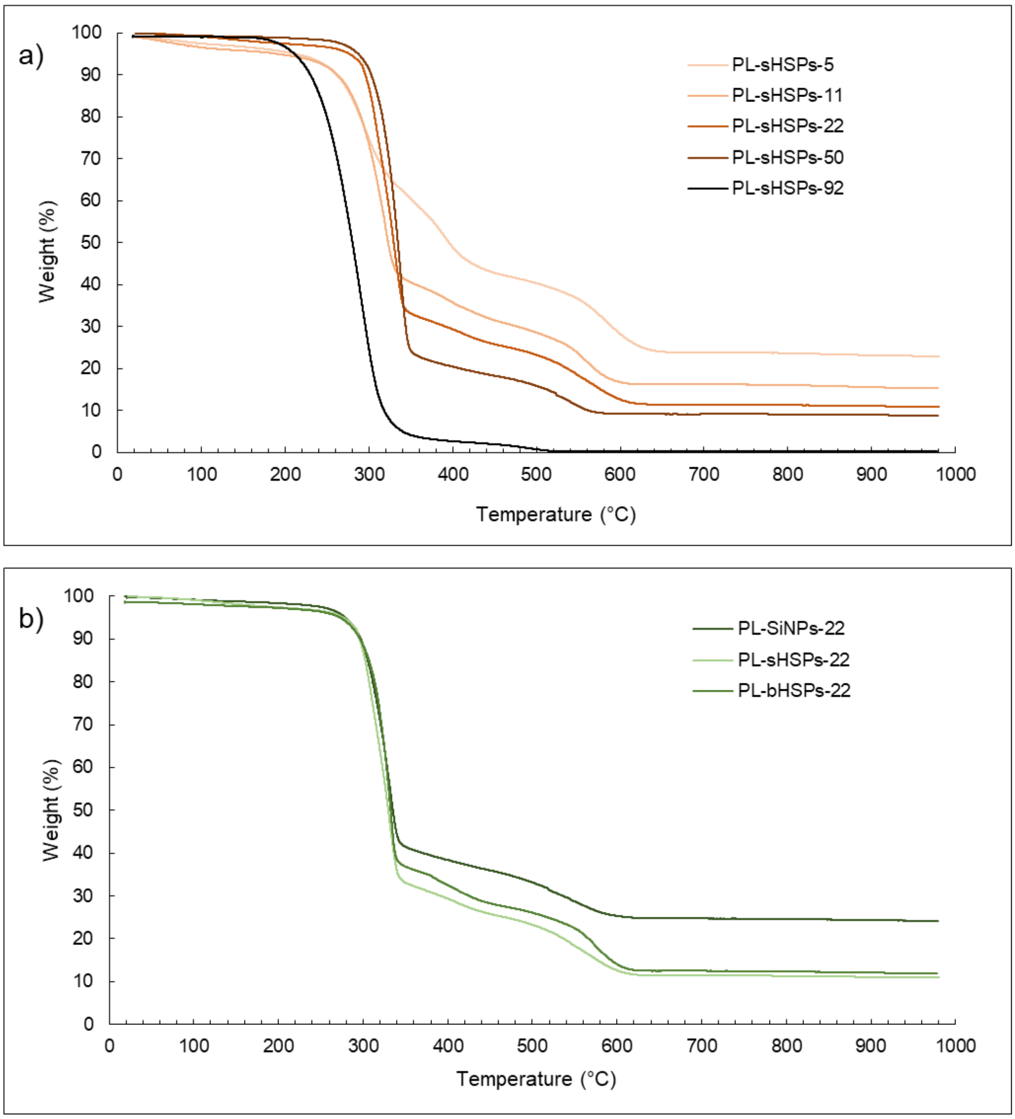

Figure S6. a) Variation of TGA profiles of PLs according to the overall PEG lengths of the EthA involved for each PL-sHSPs, b) Variation of TGA profiles of PLs depending on the nature of the silica core modified with EthA-22.

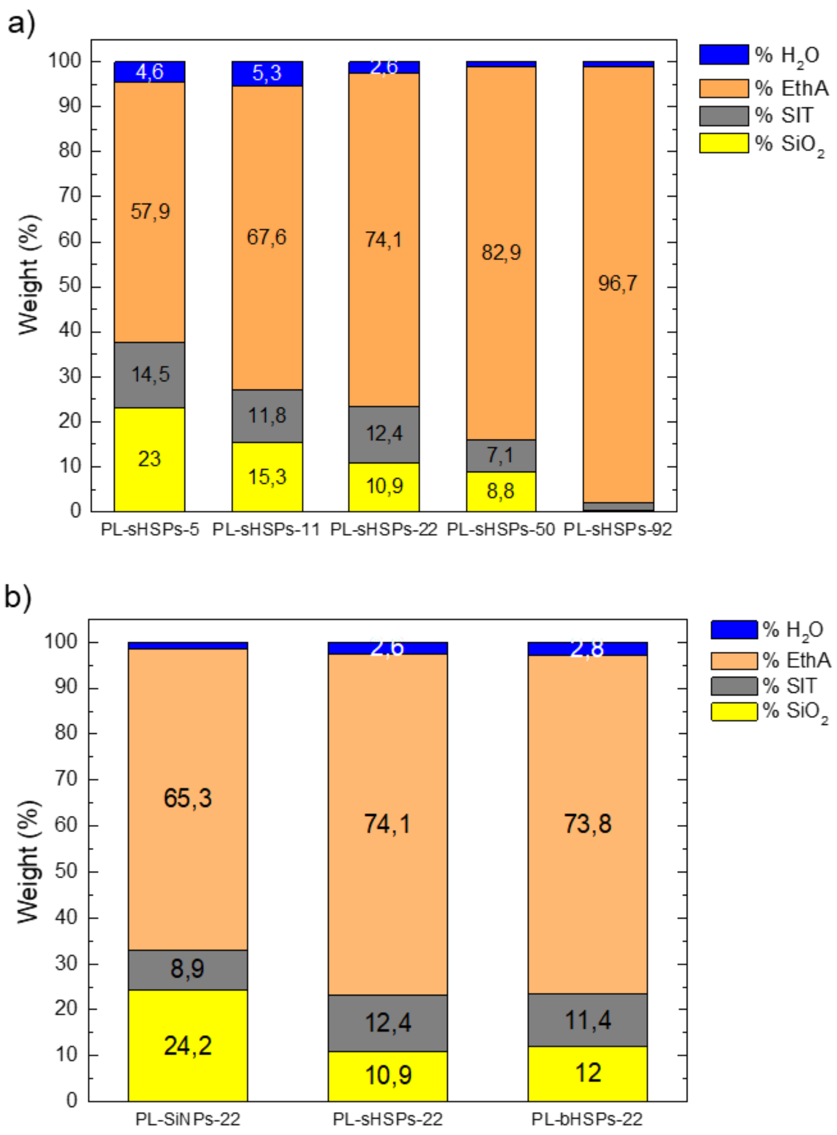

Figure S7. a) Chemical composition of PLs-sHSPs with different amine chains lengths, b) chemical composition of PLs grafted with EthA-22 on different silica spheres.

The molar ratio Amine/SiO2 displayed a non-linear behavior with the molecular weight of the amine, with an increase frome 0.32 to 0.37 between PL-sHSPs-5 and PL-sHSPs-11, a further decrease up to 0.22 for PL-sHSPs-50, and a final significant increase for PL-sHSPs-92 with a ratio exceeding 6 (Table S5). The molar ratio SIT/SiO_2 increased from 0.31 (PL-sHSPs-5) to 0.57 (PL-sHSPs-22) but decreased to 0.4 for PL-sHSPs-50. It could not be determined for PL-sHSPs-92 (no significant weight loss attributed to the SIT on the TGA trace) but it is expected to be high.

Additionally, the subequivalent Amine/SIT ratios found based on TGA measurements could mean that the pH-metry was not a sufficient method to follow the real equivalence during the neutralization of the sulfonate groups.

The ratios for PLs with EthA-22 are also given in the Table S6.

Table S5. Weight and molar ratios of Amine, SiO2 and SIT contained in PL-sHSPs with different amines.

PL-sHSPs-X	Amine/SiO_2 (w/w)	Amine/SiO_2 (m/m)	SIT/SiO_2 (w/w)	SIT/SiO_2 (m/m)	Amine/SIT (m/m)
5	2,52	0,32	0,63	0,31	1,01
11	4,42	0,37	0,77	0,38	0,95
22	6,80	0,34	1,14	0,57	0,60
50	9,42	0,22	0,81	0,40	0,55
92	460,48	6,42	-	-	-

Table S6. Weight and molar ratios of Amine, SiO2 and SIT contained in PLs with EthA-22 and different silica spheres.

PL-XX-22	Amine/SiO_2 (w/w)	Amine/SiO_2 (m/m)	SIT/SiO_2 (w/w)	SIT/SiO_2 (m/m)	Amine/SIT (m/m)
XX = SiNPs	2,70	0,14	0,4	0,18	0,74
XX = sHSPs	6,80	0,34	1,14	0,57	0,60
XX = bHSPs	6,15	0,31	0,95	0,47	0,65

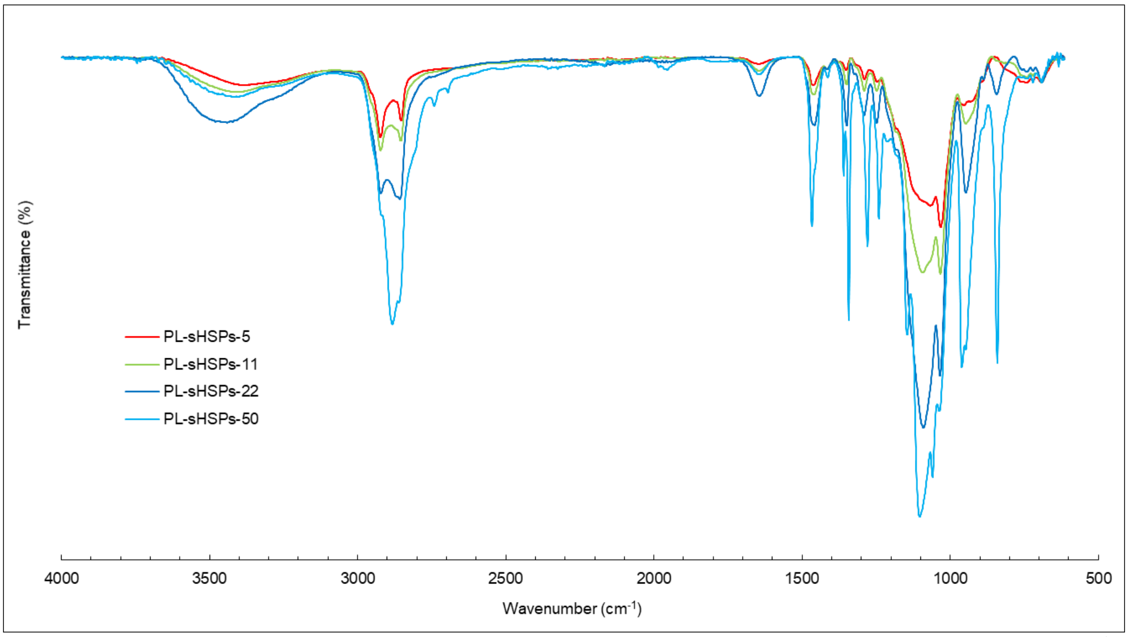

Figure S8. FTIR spectra of PL-sHSPs (n+m from 5 to 50) in the range 615-4000 cm^{-1}, normalized on the peak at 690 cm^{-1} specific of the organosilicon.

The FTIR trace of PL-sHSPs-92 could not be normalized since the proportion of amine with respect to the organosilicon was too high to see a well-defined peak at 690 cm^{-1}.

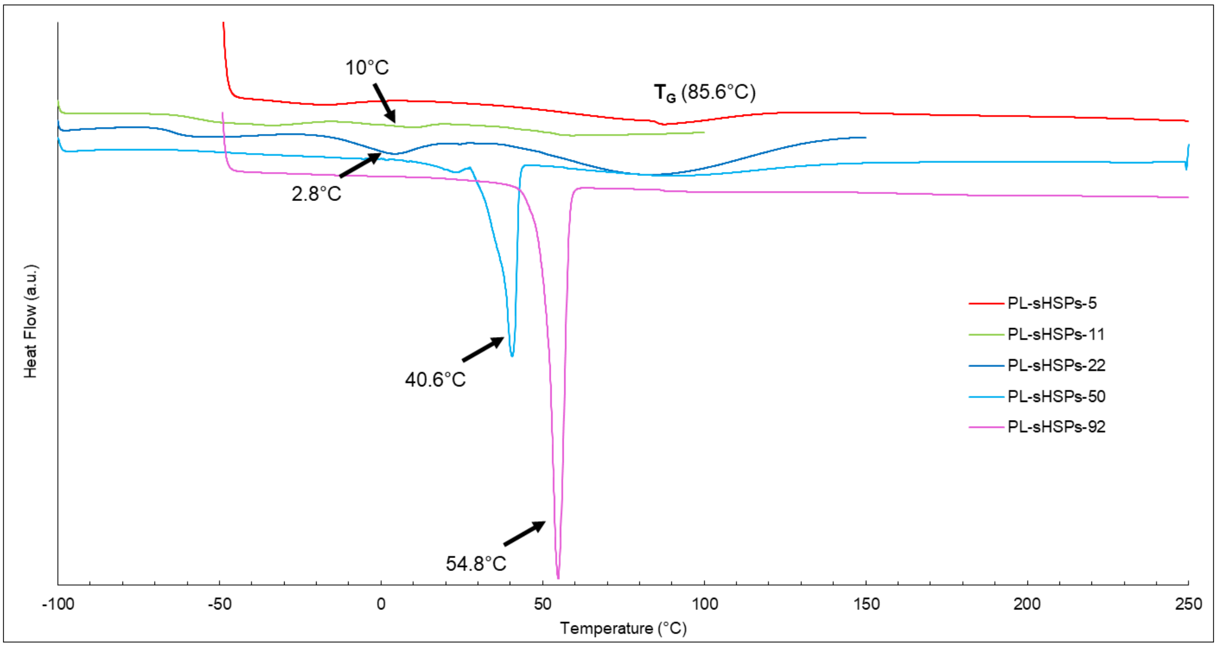

Figure S9. DSC profiles of all PL-sHSPs. The arrows indicate the melting peaks, and the values correspond to the midpoint. For PL-sHSPs-5, no melting was observed, but a glass transition was clearly visible at 85.6°C.

S4. Viscometry experiments

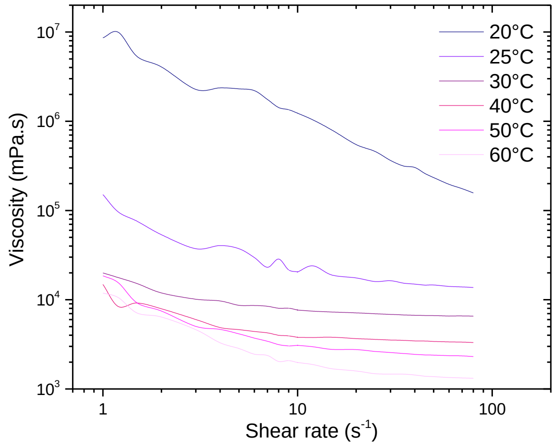

Figure S10. Viscosity of PL-sHSPs-22 plotted at different temperatures versus the shear rate (log-log scale).

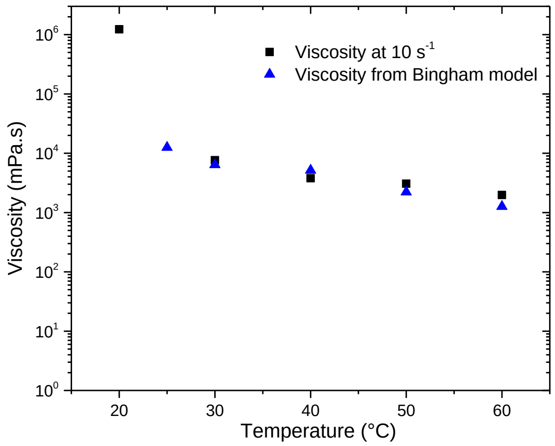

Figure S11. Viscosity of PL-sHSPs-22 at different temperatures (log-log scale), recorded at 10 s^{-1} (black squares) or extracted from a Bingham fitting of the measurements (blue triangles).

As expected, a non-Newtonian behavior was observed for the liquids along with high viscosities (Figure S9), and the curves could be modelled by a Bingham fitting (with an exception for the viscosity at 20°C that did not follow the same behavior). The values of viscosities obtained with this model (Figure S10) were close to the one obtained at 10 s^{-1} for each PL (corresponding to a shear rate at the beginning of the zone of Newtonian behavior).

S5. Contact test of PL-sHSPs-11 and PL-sHSPs-22 with water

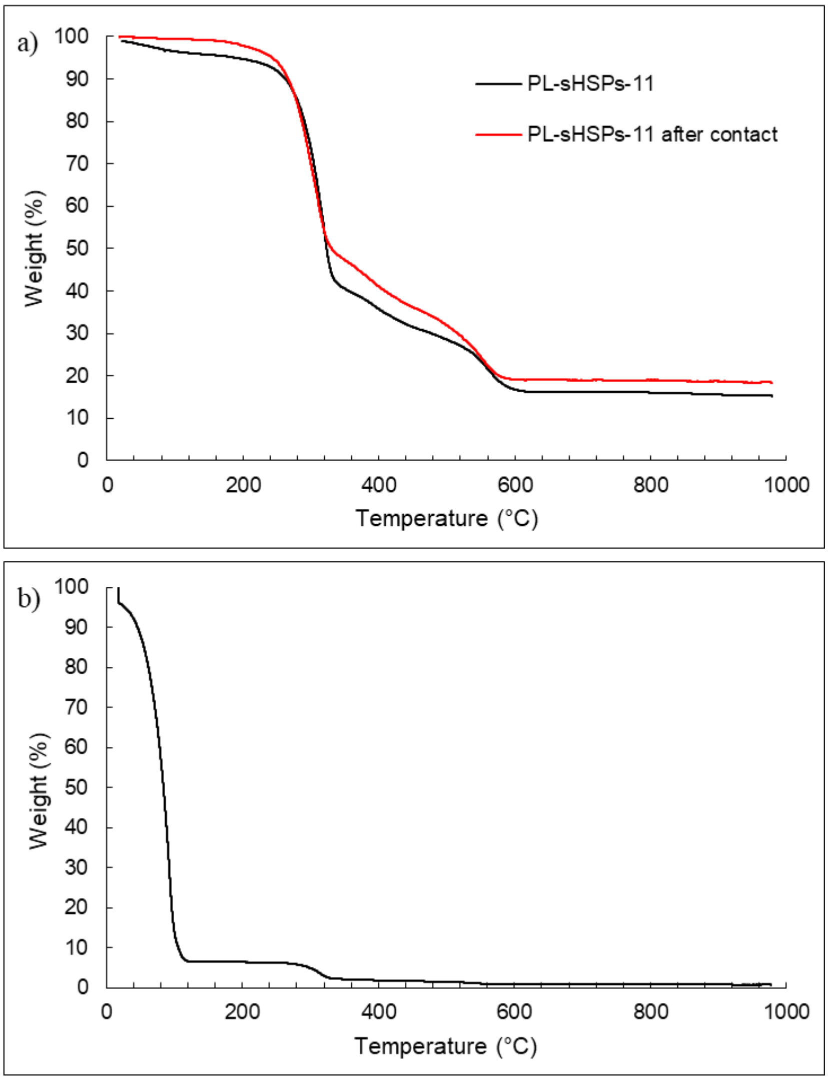

Figure S12. a) TGA of PL-sHSPs-11 before (black) and after (red) contact with water. b) TGA of the aqueous phase after contact of PL-sHSPs-22 with water in a w:w 1:20 PL:water ratio.
